# The Utility of Whole Exome Sequencing in Fetuses With Isolated Increased Nuchal Translucency

**DOI:** 10.1002/mgg3.70161

**Published:** 2025-11-29

**Authors:** Hui Wang, Caiqun Luo, Qian Geng, Xiaoxin Xu, Yang Liu

**Affiliations:** ^1^ Maternal‐Fetal Medicine Centre Shenzhen Maternity and Child Healthcare Hospital Shenzhen China; ^2^ Medical Genetics Centre Shenzhen Maternity and Child Healthcare Hospital Shenzhen China

**Keywords:** fetuses, isolated, nuchal translucency (NT)

## Abstract

**Background:**

To evaluate the utility of prenatal whole exome sequencing (WES) in cases of isolated increased nuchal translucency (NT).

**Methods:**

Retrospective analysis of the prenatal WES results in fetuses with increased NT (≥ 3.0 mm) and normal fetal anatomy, normal karyotype, and chromosomal microarray analysis (CMA). Subgroup analysis was performed based on NT measurements.

**Results:**

Diagnostic variants were identified in 3 of 118 fetuses (2.5%) with isolated increased NT (≥ 3.0 mm). The distribution of positive findings was as follows: NT 3.0–3.4 mm group (*n* = 13): 1 case (7.7%) with diagnostic genetic variants; NT ≥ 3.5 mm group (*n* = 105): 2 cases (1.9%) with diagnostic genetic variants (both in the NT 5.0–6.4 mm subgroup). Fisher's exact test (two‐tailed) showed no statistically significant differences in diagnostic yield between: NT 3.0–3.4 mm versus NT ≥ 3.5 mm (*p* = 0.298); NT 3.0–3.4 mm versus NT 5.0–6.4 mm (*p* = 1.000); NT ≥ 3.0 mm versus NT ≥ 3.5 mm (*p* = 1.000).

**Conclusion:**

Our analysis revealed a low diagnostic yield (2.5%) for prenatal WES in cases of isolated increased NT ≥ 3.0 mm. Our findings provide valuable evidence for clinical counseling, particularly when patients inquire about the likelihood of isolated findings. These data offer meaningful guidance for their decision‐making process regarding further testing options.

## Graphical abstract

In this work, we evaluated the utility of whole exome sequencing in fetuses with isolated increased nuchal translucency. Our analysis revealed a low diagnostic yield (2.5%) for prenatal WES in cases of isolated increased NT ≥ 3.0 mm. Our findings provide valuable evidence for clinical counseling.

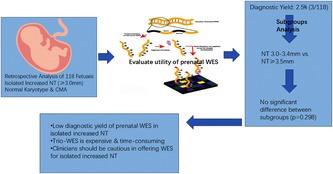

## 1 Introduction

Nuchal translucency (NT) measurement is a crucial first‐trimester ultrasound marker, routinely assessed between 11 and 14 weeks of gestation for screening fetal chromosomal abnormalities. An increased NT, defined as measurement ≥ 95th percentile, is associated with elevated risk of both fetal chromosomal abnormalities and structural malformations (American College of Obstetricians and Gynecologists, Society for Maternal‐Fetal Medicine 2020). Karyotype and CMA are routinely used to detect chromosomal abnormalities in fetuses with increased NT, with chromosomal abnormalities identified in approximately 30% of these cases (Bardi et al. 2020). Even when karyotype and CMA are normal, fetuses with increased NT remain at elevated risk of adverse outcomes, including miscarriage, intrauterine fetal death, congenital heart disease, and developmental delay (Bilardo et al. 2007, 2010; Maymon et al. 2005).

With the development of sequencing technology, prenatal exome sequencing (ES) has been used to detect monogenic disorders in fetuses with structural anomalies and negative karyotype and CMA results. In 2019, two large, prospective studies demonstrated that trio ES provided an additional diagnostic yield of 8.5% and 10.3% respectively, in fetuses with any ultrasound abnormality and normal karyotype and CMA results (Lord et al. 2019; Petrovski et al. 2019). Previous studies have demonstrated that trio ES yields an increased diagnostic rate (20%–40%) in fetuses with both increased NT and additional structural anomalies (Cao et al. 2023; Mellis, Eberhardt, et al. 2022; Mellis, Oprych, et al. 2022). In contrast, the diagnostic yield of trio ES is substantially lower (1%–4%) in fetuses with isolated increased NT (Cao et al. 2023; Di Girolamo et al. 2023; Mellis, Eberhardt, et al. 2022; Mellis, Oprych, et al. 2022; Pauta et al. 2022; Sun et al. 2023). Thus, the clinical utility and cost‐effectiveness of prenatal exome sequencing for isolated increased NT remain uncertain and require further investigation.

In this retrospective study, we analyzed prenatal WES results in fetuses with isolated increased NT and normal second‐ and third‐trimester ultrasound anatomy, and normal karyotype and CMA results. Our objectives were to determine the diagnostic yield of WES and the frequency of variants of unknown significance (VUS) in this population.

## 2 Materials and Methods

### 2.1 Ethical Compliance

This study was approved by the Ethics Committee of the Shenzhen Maternity and Child Health Hospital (No. 2018‐280). Written informed consent was obtained from all participants prior to their inclusion in the study.

### 2.2 Study Design and Participants

This retrospective study analyzed data from pregnant women who received care at Shenzhen Maternity and Child Healthcare Hospital between 2021 and 2023. Written informed consent was obtained by obstetricians. Between 2021 and 2023, a total of 10,030 pregnant women visited our hospital for invasive prenatal diagnostics. Among them, 9802 underwent CMA, and 957 underwent Trio‐WES. Following the detection of increased NT during first‐trimester NT measurement, invasive diagnostic testing was performed either via chorionic villus sampling at 11–14 weeks of gestation or amniocentesis at 16–20 weeks of gestation. Only pregnancies meeting the study's inclusion criteria subsequently underwent enrollment. The inclusion criteria were as follows: (1) Singleton pregnancies with fetuses exhibiting isolated increased NT (defined as NT ≥ 3.0 mm), regardless of whether the increased NT/NF subsequently regressed spontaneously, progressed, or remained unchanged on follow‐up ultrasound examinations, were included in the study; (2) Normal second‐ and third‐trimester ultrasound anatomy; (3) Normal fetal karyotype and CMA results.

### 2.3 G‐Banding Karyotyping and Chromosomal Microarray Analysis

All samples underwent routine G‐banding karyotyping. For CMA and WES, genomic DNA was extracted directly from uncultured samples using the QIAamp DNA Blood MiniKit (QIAGEN, Germany). Cultured cells were preserved at −80°C as a contingency measure for potential maternal cell contamination or insufficient DNA concentrations from direct extraction. The CytoScan 750 K chip (Applied Biosystems, Affymetrix) was utilized for CMA. Detected CNVs were categorized according to the joint consensus recommendation from the ACMG and the ClinGen (Riggs et al. 2020).

### 2.4 Whole Exome Sequencing (Trio‐WES)

DNA libraries were prepared using the Berry_NanoWESv2 kit and sequenced on the NovaSeq 6000/PE150 platform (Illumina). Bioinformatics analysis was performed using the Seqmax trio analysis platforms (Yun Feng Biotech, Shenzhen). Our analysis focuses on three outcomes: (1) estimate the diagnostic yield of trio‐WES in detecting pathogenic(P) or likely pathogenic (LP)genetic variants following normal karyotype and CMA results; (2) the frequency of variants of unknown significance (VUS) identified through trio‐WES; (3) the distribution of P or LP genetic variants across varying nuchal translucency (NT) measurement thresholds. Sequence variants were classified according to the ACMG/AMP guidelines (Richards et al. 2015). Only P/LP variants considered clinically relevant to increased NT were classified as positive diagnostic results. We typically do not report incidental findings identified through prenatal trio‐WES testing.

### 2.5 Grouping and Statistical Analysis

Based on NT measurements, we stratified our cohort into two main groups: NT 3.0–3.4 mm and NT ≥ 3.5 mm. The NT ≥ 3.5 mm group was further subdivided into three subgroups: 3.5–4.9 mm, 5.0–6.4 mm, and ≥ 6.5 mm.

The two‐tailed Fisher's exact test was used to compare rates of diagnostic genetic variants among the following NT groups/subgroups: NT 3.0–3.4 mm versus NT ≥ 3.5 mm, NT 3.0–3.4 mm versus NT 5.0–6.4 mm, and NT ≥ 3.0 mm versus NT ≥ 3.5 mm.

## 3 Results

Between 2021 and 2023, trio‐WES was performed for 118 pregnant women carrying fetuses with isolated increased NT and normal karyotype and CMA results. The cohort included 13 fetuses (11.0%) with NT 3.0–3.4 mm, and 105 fetuses (89.0%) with NT ≥ 3.5 mm. Among 105 fetuses with NT ≥ 3.5 mm, the distribution was as follows: 78 fetuses (74.3%) had NT measurements of 3.5–4.9 mm, 20 (19.0%) measured 5.0–6.4 mm, and 7 (6.7%) showed NT ≥ 6.5 mm (Table 1). Fetal DNA was obtained from direct amniotic fluid samples in 82 cases (69.5%), cultured amniotic fluid in 1 case (0.8%), and direct chorionic villus samples in 35 cases (29.7%).

**TABLE 1:** Comparison of Trio‐WES detection rates between groups divided by the size of NT.

NT(mm)	Number of cases	Diagnostic variants detected (%)	*p* (Fisher's exact test)
3.0–3.4	13	1 (7.7)	*p*: 0.298 (group with NT:3.0–3.4 mm and NT ≥ 3.5 mm)
≥ 3.5	105	2 (1.9)	
3.5–4.9	78	0 (0)	
5.0–6.4	20	2 (10)	*p*: 1.000 (group with NT:3.0–3.4 mm and NT:5.0–6.4 mm)
≥ 6.5	7	0 (0)	
Total (≥ 3.0)	118	3 (2.5)	*p*: 1.000 (group with NT ≥ 3.0 mm and NT ≥ 3.5 mm)

Among 118 fetuses with NT ≥ 3.0 mm, we identified diagnostic genetic variants in 3 (2.5%), incidental findings in 1 (0.8%), and VUS in 1 (0.8%) that may be associated with increased NT. The distribution of positive findings was as follows: NT 3.0–3.4 mm group (*n* = 13): 1 case (7.7%) with diagnostic genetic variants; NT ≥ 3.5 mm group (*n* = 105): 2 cases (1.9%) with diagnostic genetic variants (both in the NT 5.0–6.4 mm subgroup). Fisher's exact test (two‐tailed) showed no statistically significant differences in diagnostic yield between NT 3.0–3.4 mm and NT ≥ 3.5 mm (*p* = 0.298); NT 3.0–3.4 mm and NT 5.0–6.4 mm (*p* = 1.000); NT ≥ 3.0 mm and NT ≥ 3.5 mm (*p* = 1.000) (Table 1).

Detailed information about genetic diagnostic findings:

Three cases with positive diagnostic genetic variants were identified (Table 2). Case 65 presented with an NT of 3.0 mm and carried a heterozygous *NF1* variant (NM_001042492.3):c.3610C>T (p.Arg1204Trp), inherited from the father. Post‐test counseling revealed that the father had multiple café‐au‐lait macules, consistent with neurofibromatosis type 1 (NF1). This variant was classified as likely pathogenic (LP) according to ACMG guidelines (PS45_Moderate + PM5 + PS3_Supporting + PM2_Supporting + PP3). Case 93 exhibited an NT of 6.0 mm and harbored a heterozygous *RIT1* variant (NM_006912.6):c.67A>G (p.Lys23Glu), inherited from the mother, who declined further clinical assessment for Noonan syndrome but showed no overt features. The variant was classified as LP according to ACMG guidelines (PM6 + PM5 + PS4_Supporting+PP3_Moderate+PM2_Supporting). Case 117 had an NT of 5.6 mm and carried a heterozygous *EPHB4* variant (NM_004444.5):c.2512C>T (p.Arg838Trp), inherited from the mother, who displayed multiple small (1–2 cm) capillary malformations. This variant was also classified as LP according to ACMG guidelines (PS4_Moderate + PS3_Supporting + PP3_Moderate+PP1).

**TABLE 2:** Clinical and molecular characteristics of fetuses with diagnostic genetic variants.

Case ID	CRL/NT (mm)	Variants	Zygosity	Disease	ACMG classification	Outcomes
65	58.1/3.0	*NF1*, NM_001042492.3: c.3610C>T (p.Arg1204Trp), [Pat]	Het	Neurofibromatosis, type 1 (OMIM: #162200)	LP (PS4_Moderate+PM5 + PS3_Supporting+PM2_Supporting+PP3)	Live birth
93	57.8/6.0	*RIT1*, NM_006912.6: c.67A>G (p.Lys23Glu), [Mat]	Het	Noonan syndrome 8 (OMIM: #615355)	LP (PM6 + PM5 + PS4_Supporting+PP3_Moderate+PM2_Supporting)	Normal growth and development were observed at 12 months of age
117	68.3/5.6	*EPHB4*, NM_004444.5: c.2512C>T (p.Arg838Trp), [Mat]	Het	*EPHB4*‐associated vascular malformation spectrum (OMIM: #618196, 617300)	LP (PS4_Moderate + PS3_Supporting + PP3_Moderate + PP1)	At 7 months of age, growth and development were normal, echocardiography revealed a left‐to‐right shunt at the midportion of the atrial septum, no seizures were observed
		*SCN1A*, NM_001165963.4: c.3734G>A(p.Arg1245Gln), [Mat]	Het	*SCN1A* seizure disorders (OMIM: #619317, 607208, 604403, 609634)	LP (PP3_Moderate + PS2_Supporting + PS4_Moderate + PM2_Supporting + PP2)	

An incidental finding was identified in Case 117, which presented with an NT of 5.6 mm. Alongside the *EPHB4* variant, this case carried a heterozygous *SCN1A* variant (NM_001165963.4):c.3734G>A (p.Arg1245Gln), inherited from the mother. The mother reported no personal or family history of *SCN1A*‐related seizure disorders. This variant was classified as likely pathogenic (LP) according to ACMG guidelines (PS422_Moderate + PS2_Supporting + PP2 + PP3_Moderate + PM2_Supporting).

Among the variants of uncertain significance (VUS) potentially associated with increased NT (Table 3), Case 82 presented with an NT of 4.4 mm and compound heterozygous *FBP1* variants. The first variant (NM_000507.4):c.490G>A (p.Gly164Ser), inherited from the father, was classified as pathogenic (P) according to ACMG guidelines (PM3_VeryStrong + PP3_Moderate + PM2_Supporting). The second variant (NM_000507.4):c.721T>C (p.Tyr241His), inherited from the mother, was classified as VUS according to ACMG guidelines (PP3_Moderate+PM2_Supporting).

**TABLE 3:** Clinical and molecular characteristics of a fetus carrying VUS variants with potential clinical relevance to increased NT.

Case ID	CRL/NT (mm)	Variants	Zygosity	Disease	ACMG classification	Outcomes
82	61.3/4.4	*FBP1*, NM_000507.4: c.490G>A (p.Gly164Ser), [Pat]; *FBP1*, NM_000507.4: c.721T>C (p.Tyr241His), [Mat]	Compound heterozygosis	Fructose‐1,6‐bisphosphatase deficiency (OMIM: #229700)	P (PM3_VeryStrong + PP3_Moderate + PM2_Supporting); VUS (PP3_Moderate + PM2_Supporting)	Ultrasound at 32 weeks gestation showed no significant abnormalities. The patient subsequently declined further follow‐up

## 4 Discussion

In this retrospective study of 118 fetuses with isolated increased NT and normal karyotype and CMA results, trio‐WES revealed a low diagnostic yield (2.5%), consistent with prior research (Mellis, Eberhardt, et al. 2022; Mellis, Oprych, et al. 2022; Cao et al. 2023; Di Girolamo et al. 2023).

There is no consensus about the optimal threshold for increased NT. The most commonly used cutoff value is NT ≥ 3.5 mm which generally reflects an NT above the 99th percentile at any CRL between approximately 11 and 14 weeks or NT ≥ 3.0 mm (above the 95th percentile) (American College of Obstetricians and Gynecologists and Society for Maternal‐Fetal Medicine 2020). An NT measurement above 3.5 mm is commonly used as the threshold for recommending prenatal Trio‐WES. However, data on the diagnostic yield of Trio‐WES in fetuses with isolated increased NT (ranging from 3.0 to 3.4 mm) remain limited. To date, only a few studies have investigated this subgroup, reporting diagnostic rates between 3% and 12%, which is consistent with our findings (7.7%). Notably, the majority of identified pathogenic variants were associated with RASopathies, further corroborated by our results (Case 65) (Sun et al. 2023; Jing et al. 2025). Due to the small sample sizes in both the existing literature and our study, the true diagnostic rate of prenatal Trio‐WES in fetuses with NT measurements of 3.0–3.4 mm remains uncertain. Further large‐scale studies are needed to establish a more precise diagnostic yield in this population.

Among the three fetuses with diagnostic variants, two (Case 65, Case 93) carried RASopathy‐associated genetic variants. This finding aligns with the established association between increased NT and RASopathies, where NT enlargement represents the most common prenatal manifestation (Chen 2022; Stuurman et al. 2019). Given that two of the findings were RASopathies that could have been detected on a RASopathy panel, perhaps this was an alternative option for fetuses with isolated increased NT. This would decrease the issues alluded to with VUSes and finances compared to Trio‐WES. Similar findings have been reported in previous studies (Sun et al. 2023; Mellis, Eberhardt, et al. 2022; Mellis, Oprych, et al. 2022).

Although the *NF1* gene is classified as a RASopathy‐associated gene, the prenatal phenotypic manifestations of *NF1* variants remain poorly characterized. In our study, we identified a likely pathogenic (LP) *NF1* variant in Case 65, which presented with an NT of 3.0 mm. This finding aligns with a previously reported *NF1* variant (NM_001042492.2: c.2239A>G) in a fetus with isolated increased NT (3.7 mm) (Cao et al. 2023). Based on these observations, we consider the *NF1* variant in Case 65 to be a clinically significant diagnostic finding. Regarding *RIT1* gene variants associated with Noonan syndrome, multiple reports have documented their occurrence in fetuses with increased NT, hydrops fetalis, and other ultrasound features suggestive of Noonan syndrome (Qiu et al. 2022).

*EPHB4* gene variants have been reported in multiple fetuses presenting with increased NT and/or hydrops fetalis (Mellis, Eberhardt, et al. 2022; Mellis, Oprych, et al. 2022; Vanden et al. 2024). In Case 93, we identified an *EPHB4* missense variant located within the tyrosine kinase domain (AA: 615–899), a region known to be enriched for pathogenic missense variants (Zeng et al. 2019). The phenotype of *EPHB4* has high intra‐ and interfamilial variability and can be associated with a spectrum of phenotypes ranging from subtle features of lymphatic dysfunction to lethal nonimmune hydrops fetalis. Notably, Vanden et al. (2024) reported a missense variant (*EPHB4* c.2791G>A, p.Ala931Thr) in the adjacent SAM domain, identified in a fetus with an isolated large NT that resolved spontaneously—a presentation highly similar to our Case 93.

VUS variants are challenging results for clinicians, patients, and laboratories because of the uncertainty for diagnoses, especially in prenatal diagnosis. These results may frustrate clinicians and can be misinterpreted and bring long‐term anxiety to patients (Chen et al. 2023). In this study, we reported a VUS variant in Case 82 (Table 3). While the prenatal phenotype of fructose‐1,6‐bisphosphatase deficiency is not well characterized, we hypothesize that the *FBP1* gene variant detected in Case 82 could explain the increased nuchal translucency, as inborn errors of metabolism are known to cause nonimmune hydrops fetalis (Sparks et al. 2020).

Because of the limited sample size of the group with NT: 3.0–3.4 mm, the comparison between the groups may be too skewed. Due to insufficient postnatal follow‐up data, we cannot definitively confirm whether the included cases represent true isolated increased NT. Since subsequent progression of NT/NF was not assessed in these cases, the diagnostic rate may have been underestimated in fetuses with persistent NT/NF thickening.

## 5 Conclusion

To our knowledge, this is one of the relatively large sample size cohort studies investigating isolated increased NT, revealing a low diagnostic rate using Trio‐WES. Prenatal Trio‐WES currently remains relatively expensive and time‐consuming. Improper use can cause serious financial burden and anxiety for pregnant women. Our findings provide valuable evidence for clinical counseling, particularly when patients inquire about the likelihood of isolated findings. These data offer meaningful guidance for their decision‐making process regarding further testing options.

## Author Contributions

Yang Liu and Hui Wang designed the study and drafted the manuscript. Caiqun Luo, Qian Geng, Xiaoxin Xu acquired and interpreted the data. Yang Liu is the corresponding author of this manuscript. All authors read and approved the final manuscript.

## Funding

Sanming Project of Medicine in Shenzhen (No. SZSM202311005).

## Conflicts of Interest

The authors declare no conflicts of interest.

## Data Availability

The datasets used and/or analysed during the current study are available from the corresponding author on reasonable request.
